# Nontraumatic Paraureteral Urinoma in a Cat with Urolithiasis

**DOI:** 10.3390/ani12212934

**Published:** 2022-10-26

**Authors:** Marco Tabbì, Claudia Rifici, Luca Cicero, Francesco Macrì, Giuseppe Mazzullo, Alessandra Sfacteria, Giovanni Cassata, Massimo De Majo, Simona Di Pietro

**Affiliations:** 1 Department of Veterinary Sciences, University of Messina, 98168 Messina, Italy; 2 Istituto Zooprofilattico Sperimentale della Sicilia “A. Mirri”, Via Gino Marinuzzi 3, 90100 Palermo, Italy

**Keywords:** kidney disease, feline urinoma, ultrasound-guided percutaneous injection, ureteral calculi

## Abstract

**Simple Summary:**

In this study, the clinical and diagnostic findings of a well-encapsulated para-ureteral urinoma associated with urinary-tract stones in a critical feline patient are reported. Due to the worsening of the cat’s health, to better define the extent and genesis of the lesion, a positive contrast radiographic study was performed by means of an ultrasound-guided percutaneous injection of a contrast medium into the lesion, which highlighted a wide and homogeneous radiopaque area in the left retroperitoneal space. These findings were confirmed with a postmortem examination, corroborating the diagnostic suspicion of urinoma.

**Abstract:**

Urinoma is an encapsulated collection of urine due to a disruption in the collection system of the urinary tract. This condition is rarely reported in veterinary medicine. The aim of this study is to describe the clinical and diagnostic findings of a well-encapsulated paraureteral urinoma associated with urinary tract stones in a critical feline patient. The ultrasound examination of the abdomen revealed a well-defined collection of anechoic fluid containing the left kidney in the retroperitoneal space, while the radiographic examination revealed a loss of soft tissue contrast in the lumbar region. Both techniques confirmed the presence of stones in the bladder. Following the ultrasound-guided drainage of the lesion, the nature of the fluid was also confirmed to be urine. Due to the worsening of the cat’s health, to better define the extent and genesis of the lesion, a positive contrast radiographic study was performed by means of an ultrasound-guided percutaneous injection of a contrast medium into the lesion, which highlighted a wide and homogeneous radiopaque area in the left retroperitoneal space. These findings were confirmed with a postmortem examination, corroborating the diagnostic suspicion of urinoma. The percutaneous contrast inoculation performed in this critically ill patient plays a role in the diagnostic process to reach a final diagnosis in cases in which the patient’s clinical condition does not allow for performing an excretory positive contrast study.

## 1. Introduction

Urinoma or uriniferous pseudocyst is an encapsulated collection of urine that leaks from the urinary tract due to an interruption in the urinary collection system at any level, from the calix to the urethra [1,2,3]. In human medicine, urinoma is widely reported because of blunt or penetrating trauma, fibrosis, or a surgery of the retroperitoneal space, intra-abdominal neoplasms, and urinary-tract obstruction by uroliths [1,4,5]. In veterinary medicine, urinoma is an uncommon condition with few reports described in small and large animals [6,7,8]. In cats, urinomas are often described as post-traumatic in nature. The radiography, ultrasonography, excretory urography, and computed tomography of pseudocysts and the urinary tract were used to diagnose urinomas [8,9,10,11,12]. This study describes the clinical and diagnostic findings of a well-encapsulated paraureteral urinoma associated with urinary-tract stones in a critical feline patient.

## 2. Case Description

A 5-year-old neutered domestic shorthaired male cat was presented with clinical signs of lethargy, anorexia, and anuria during the last 24 h. The cat had an indoor lifestyle and had a chronic clinical history of urolithiasis, with recurrent episodes of hematuria, pollakiuria, and dysuria/stranguria. The clinical examination revealed sensory depression, moderate dehydration (6–8%) attested by a moderate loss of skin turgor, dry and pale mucous membranes, weak rapid pulses, and enophthalmos. Increased capillary refill time (>2 s), decreased rectal temperature (37.5 °C), rapid breathing, and abdominal distension with pain on palpation were also observed. Written informed consent was obtained from the owner to perform the required clinical investigations. A peripheral venous blood sample was taken to perform a complete blood count and a biochemical examination (azotemia, creatinine, transaminases, total proteins, and alkaline phosphatase). Mild neutrophilic leukocytosis with a left shift (10,330/μL, range from 1480 to 10,290/μL), thrombocytopenia (102,000/μL, range 151,000–600,000/μL), increased serum creatinine (10.4 mg/dL, range from 0.8 to 2.4 mg/dL), urea (>130 mg/dL, range from 16 to 36 mg/dL), and hypoproteinemia were observed. Intravenous fluid therapy was performed by administering isotonic crystalloid solutions, and pain medication with methadone was started (Semfortan 10 mg/mL, Eurovet Animal Health B.V AE Bladel, The Netherlands; 0.2 mg/kg IM).

**Figure 1:**
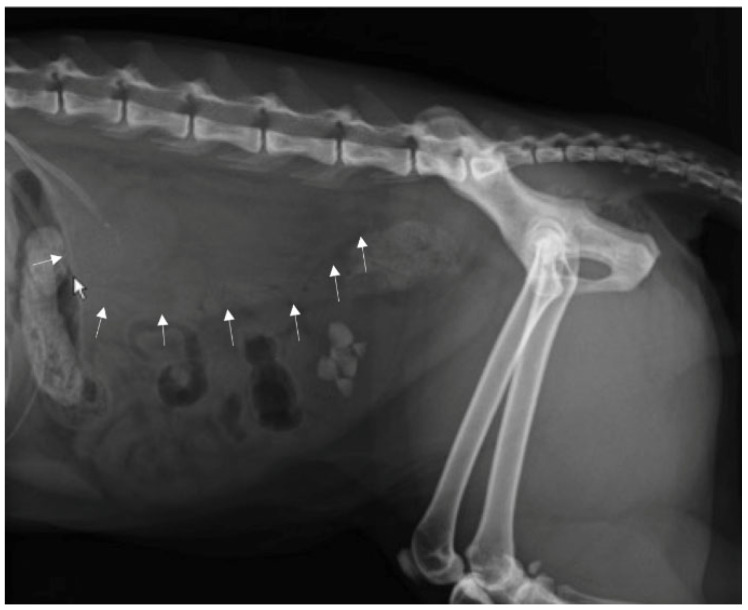
Lateral radiograph of the abdomen: loss of contrast in the lumbar region (arrows) and a presence of uroliths in urinary bladder.

A direct radiographic study of the abdomen with orthogonal projections was performed, using a radiographic system (Morpheus, Gieffe Elettromedicali, Misterbianco (Catania, Italy) and a digital radiography system (Fujifilm Medical Systems, Milan, Italy). This examination showed a loss of soft tissue contrast in the lumbar region, and radiopaque stones in the urinary bladder (Figure 1).

**Figure 2:**
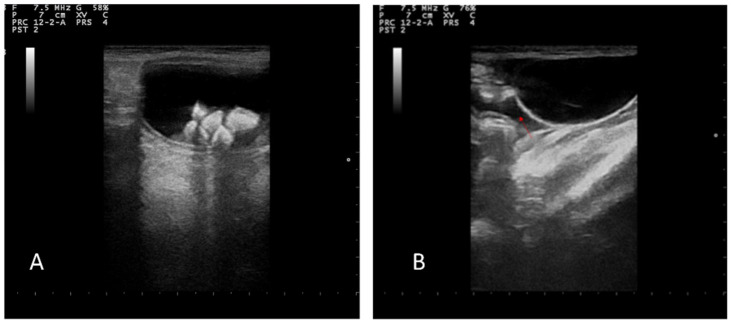
(**A**) Ultrasonographic images of urinary bladder lithiasis. (**B**) Free fluid in abdomen (red arrow).

The abdominal ultrasound examination was performed using a device equipped with 5–8 MHz microconvex and 10–12 MHz linear probes (MyLab Vet 40, Esaote, Italy). Hyperechoic areas in the bladder with a posterior shadow were observed, confirming the presence of previously diagnosed uroliths; the bladder wall appeared to be intact with normal stratification. A modest amount of free fluid was observed in the peritoneum (Figure 2A,B).

**Figure 3:**
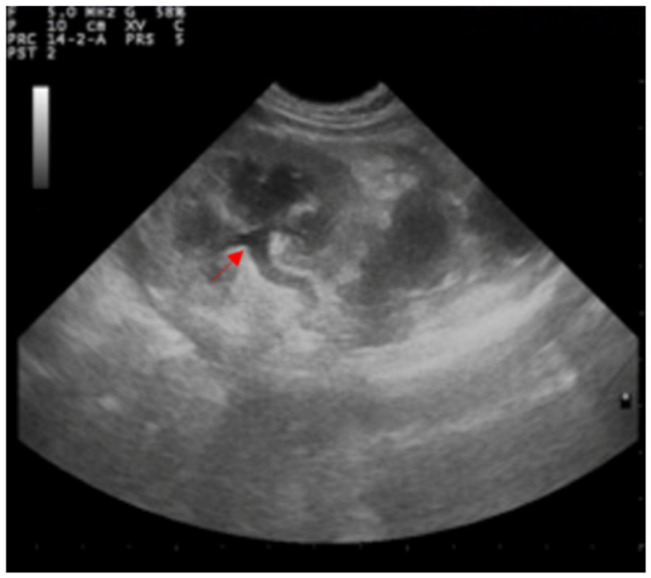
Ultrasound examination of the left kidney showing the dilatation of the renal pelvis and ureter (red arrow).

Both kidneys appeared to be enlarged with a dilated pelvis (4.5 mm), preserved echo structure, and intact renal capsule. Both ureters appeared to be dilated (5.6 mm), although the entire length of the left ureter was well-visible in contrast to the contralateral (Figure 3).

**Figure 4:**
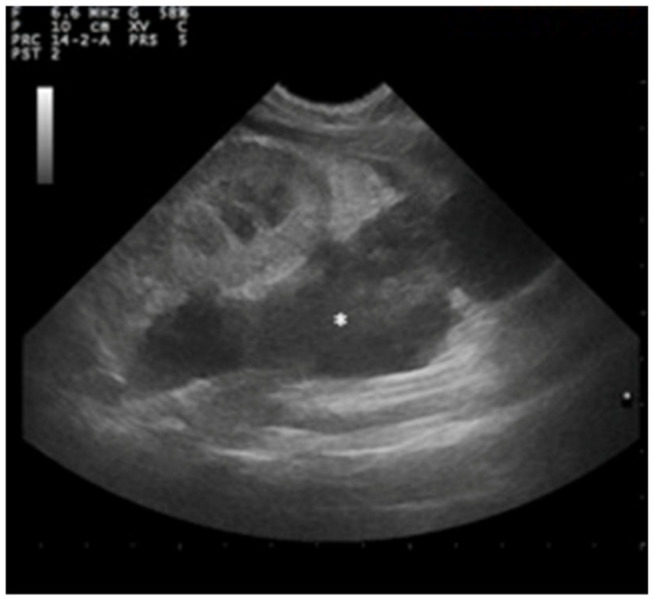
Ultrasound examination of left kidney showing anechogenic area in the left hemiabdomen (asterisk) containing small areas of echogenic tissue attributable to intraperitoneal fat.

A large perirenal collection of anechoic fluid containing small areas of echogenic tissue attributable to intraperitoneal fat was observed in the left hemiabdomen. A capsule surrounding the lesion showed an inhomogeneous pattern with no evident stratigraphy (Figure 4).

To reach a diagnosis, it was not possible to perform intravenous excretory contrast urography due to the impaired renal function; so, ultrasound-guided drainage and a percutaneous contrast medium injection on the sedated subject were performed. The cat was placed in right lateral decubitus, the skin of the renal region between the hypochondrium and the left sublumbar area was shaved and aseptically prepared for surgery, and a longitudinal and transverse preliminary scan of the kidney was performed with a high-frequency linear probe (13 MHz). Using a 8.5–10 MHz microconvex probe, an ultrasound-guided insertion of a 22-gauge spinal needle into the lesion was performed through the abdominal wall caudally to the kidney at the level of the L4–L5 intervertebral space. Then, after removing the stylet, and connecting a three-way stopcock and an extension set, the ultrasound-guided percutaneous drainage of the collection was performed by connecting a 5 mL syringe and aspiring its contents by applying gentle negative pressure. A nonionic, low-osmolar iodinated contrast medium (Iopamiro^®^ Bracco Imaging S.r.l., Milan, Italy) was injected with a volume of 5 mL, equal to the amount of fluid previously drained from the lesion.

**Figure 5:**
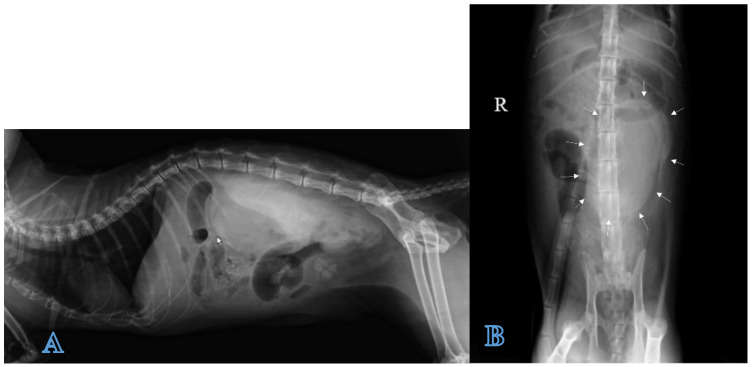
Positive contrast X-ray exam of the abdomen: (**A**) Left lateral and (**B**) ventrodorsal projections. Contrast medium that borders the fluid collection is shown in arrows.

Positive contrast radiographs in the lateral and ventrodorsal projections immediately after the injection of the contrast agent allowed for defining the extent of the lesion, outlining the fluid collection as a large well-marginated homogeneous radiopaque area in the left retroperitoneal space and differentiating it from ascite (Figure 5A,B).

The analysis of fluid collected with centesis strongly suggested the urinary nature of fluid (creatinine: 20.18 mg/dL and potassium: 5.35 mEq/L). Urethral rupture with an urinoma formation was suspected.

The patient was hospitalized; intravenous fluid therapy with Ringer’s lactate solution (Ringer Lattato^®^ S.A.L.F., Cenate Sotto, Bergamo, Italy, 3 mL/kg/h) was performed, and a surgical procedure was planned. After 24 h, the cat’s medical condition dramatically dropped and it died. In agreement with the owner, a necropsy and histological examination were performed.

**Figure 6:**
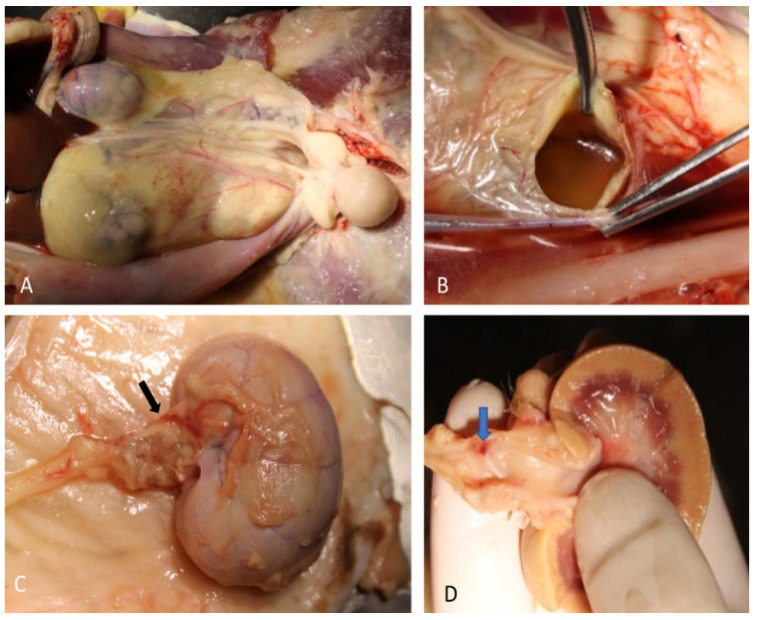
Macroscopic appearance of urinoma, (**A**) before and (**B**) after the incision. (**C**) Gross aspect of the left kidney and ureter with dilated pelvis (black arrow). At a longitudinal section of the left kidney, a pale parenchyma and an evident pyeloureteral ectasia were observed; a bright red lenticular area corresponding to the ureter rupture point (blue arrow) was observed (**D**).

The postmortem examination showed a modest peritoneal collection of dense, yellowish, odorless fluid. After displacing the intestinal loops, in the left retroperitoneal space, an 11 × 5 cm ovoid cystic formation was detected (Figure 6A). The formation was adherent to the abdominal wall, strongly attached to the ventral sublumbar musculature, adherent to the capsular surface of the left lateral liver lobe, and extending towards the urinary bladder. Its incision revealed a collection of fluid with a strong urine smell (Figure 6B). The complete opening of the formation revealed that it totally englobed the left kidney, and the proximal portion of the ureter that was irregularly dilated particularly at the pelvic trait (Figure 6C). A longitudinal section of the kidney revealed a pale appearance of the parenchyma with yellowish chromatic shades and evident pyeloureteral ectasia. A 1.5 mm bright red lenticular area, along with an intense inflammatory reaction of the ureteral wall and periureteral tissues was also detected (Figure 6D). The opening of the bladder confirmed the presence of lithiasis; the remaining body tissues and organs were unremarkable.

The microscopic examination of the urinary sediment showed the presence of struvite crystals; the analysis of the mineral composition of uroliths confirmed that they consisted of struvite.

**Figure 7:**
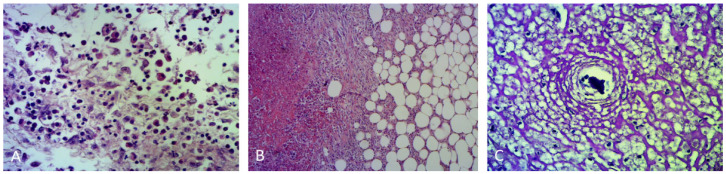
(**A**) Histological examination of the cyst wall showed fibroblasts, neutrophils and cell debris. (**B**) Sections of ureter and renal pelvis showed intense fibroblastic reaction and necrosis; (**C**) neutrophils surrounding debris of lithiasis deposits were also observed. HE, 10×.

A histological evaluation of the cyst wall showed a dense extensive streaming sheet of fibroblasts, neutrophils, and cell debris (Figure 7A–C).

A histological examination of the ureter and renal pelvis confirmed the presence of an inflammatory reaction of the ureteral wall and a thickening of the renal pelvis. The section showed adipose tissue surrounding the intense fibroblastic reaction and a copious area of necrosis; moreover, intense inflammation mainly characterized by neutrophils, which often surrounds the debris of lithiasis deposits, was found (Figure 7).

These pathological findings support the suspected diagnosis of uriniferous pseudocysts.

## 3. Discussion

Uriniferous pseudocysts are a rare disease in companion animals, with some reports available in feline medicine as post-traumatic lesion, and a study in which a spontaneous ureteral rupture due to renal calculi was suspected [9,10,11,12]. The urinoma is derived from the perirenal fat, due to the urine inducing lipolysis within 24 h. In humans, the fibrous sac around collected urine is composed of fibroblastic and round cell infiltration of the necrotic fat and adjacent renal fascia [4,9]. The azotemia is largely postrenal; after the surgical removal of the source of extravasation and fluid drainage, recovery is good.

In dogs and cats, radiography and ultrasonography are useful diagnostic tools for documenting ureteral rupture and urinoma formation, although excretory intravenous urethrography and computed tomography still remain the gold-standard methods. In fact, a contrast study is necessary to confirm the location of the leakage and to plan surgical therapy [12]. Nevertheless, an intravenous contrast agent is a nephrotoxic risk factor in patients whose renal function is already compromised [13,14]. In this study, the cat was presented in a shock condition (hypothermia, dehydration, prolonged capillary refill time, severe azotemia), rendering contrast radiography and excretory urography contraindicated [15]. The cat’s critical condition forced us to inject an ultrasound-guided percutaneous contrast medium into the lesion as an emergency diagnostic approach, supporting a diagnosis of a uriniferous pseudocyst. This imaging modality allows for evaluating the exact location and extent of the lesion when a contrast medium intravenous administration is not recommended.

In this case report, an ureteral obstruction due to lithiasis with ureter rupture and the consequent formation of the pseudocyst was suspected. This hypothesis was supported by the absence of a referred traumatic history and by the necropsy examination, which confirmed the presence of bladder stones already highlighted by radiology, and urolithic debris deposits identified with the histological exam; it showed no other causes of perforation of the ureter. In addition to the obstruction of the ureter by the stones, hydronephrosis may be due to the blocking of urinary excretion, secondary to the high pressure inside the urinoma.

The combination of imaging, and clinical and laboratory findings led us to the suspected diagnosis. The ultrasound-guided percutaneous contrast inoculation helped in radiographically outlining the well-encapsulated fluid collection and differentiating it from ascite, playing a role in the decision to propose the surgery in order to find the source of extravasation and to perform the fluid drainage, as recommended by previous studies [8,10,12]. The early death of patient did not allow for performing it.

Feline uriniferous pseudocysts are rarely associated with urinary-tract stones, as evidenced by a lack of reported clinical cases. Clinical signs are often only suggestive of a nonspecific postrenal disorder; therefore, it is necessary to combine different diagnostic techniques to achieve the final diagnosis. The percutaneous contrast inoculation performed in this critically ill patient played a role in the diagnostic process to reach a final diagnosis, and can be extended to those cases in which the patient’s clinical condition does not allow for performing an excretory positive contrast study.

## 4. Conclusions

Although it is a rare condition, feline paraureteral urinoma should be considered to be a potential complication in cats affected by urolithiasis.

## Data Availability

The data presented in this study are available on request from the corresponding author.

## References

[1] Titton R.L., Gervais D.A., Hahn P.F., Harisinghani M.G., Arellano R.S., Mueller P.R. (2003). Urine leaks and urinomas: Diagnosis and imaging-guided intervention. Radiographics.

[2] McGeady J.B., Breyer B.N. (2013). Current epidemiology of genitourinary trauma. Urol. Clin. N. Am..

[3] Phillips B., Holzmer S., Turco L., Mirzaie M., Mause E., Mause A., Person A., Leslie S.W., Cornell D.L., Wagner M. (2017). Trauma to the bladder and ureter: A review of diagnosis, management, and prognosis. Eur. J. Trauma Emerg. Surg..

[4] Healy M.E., Teng S.S., Moss A.A. (1984). Uriniferous pseudocyst: Computed tomographic findings. Radiology.

[5] Goldwasser J., Wahdat R., Espinosa J., Lucerna A. (2018). Urinoma: Prompt diagnosis and treatment can prevent abscess formation, hydronephrosis, and a progressive loss of renal function. Case Rep. Emerg. Med..

[6] Tidwell A.S., Ullman S.L., Schelling S.H. (1990). Urinoma (para-ureteral pseudocyst) in a dog. Vet. Radiol..

[7] Lopez A., Lofstedt J., Burton S. (1995). Urinoma (para-ureteral pseudocyst) in a postparturient cow. Can. Vet J..

[8] Manzini M., Crisi P.E., Del Signore F., Torre V., Stehlik L., Tamburro R., Vignoli M. (2020). Post-traumatic urinoma in two cats: Imaging diagnosis. Veterinární Med..

[9] Geel J.K. (1986). Perinephric extravasation of urine with pseudocyst formation in a cat. J. S. Afr. Vet. Assoc..

[10] Bacon N.J., Anderson D.M., Baines E.A., White R.A.S. (2002). Post-traumatic para-ureteral urinoma (uriniferous pseudocyst) in a cat. Vet. Comp. Orthop. Traumatol..

[11] Moores A.P., Bell A.M., Costello M. (2002). Urinoma (para-ureteral pseudocyst) as a consequence of trauma in a cat. J. Small Anim. Pract..

[12] Worth A.J., Tomlin S.C. (2004). Post-traumatic paraureteral urinoma in a cat. J. Small Anim. Pract..

[13] Berent A.C. (2011). Ureteral obstructions in dogs and cats: A review of traditional and new interventional diagnostic and therapeutic options. J. Vet. Emerg. Crit. Care.

[14] D’Ovidio D., Pirrone F., Donnelly T.M., Greco A., Meomartino L. (2020). Ultrasound-guided percutaneous antegrade pyelography for suspected ureteral obstruction in 6 pet guinea pigs (*Cavia porcellus*). Vet. Q..

[15] Feeney D.A., Johnston G.R., Thrall D.E., Saunders W.B. (1998). The kidneys and ureters. Textbook of Veterinary Diagnostic Radiology.

